# Can High-Intensity Functional Suspension Training over Eight Weeks Improve Resting Blood Pressure and Quality of Life in Young Adults? A Randomized Controlled Trial

**DOI:** 10.3390/ijerph16245062

**Published:** 2019-12-12

**Authors:** Florian A. Engel, Ludwig Rappelt, Steffen Held, Lars Donath

**Affiliations:** 1 Institute of Sport and Sport Science, Department Movement and Training Science, Heidelberg University, 69120 Heidelberg, Germany; 2 Department of Intervention Research in Exercise Training, German Sport University Cologne, 50933 Köln, Germany; l.rappelt@dshs-koeln.de (L.R.); s.held@dshs-koeln.de (S.H.); l.donath@dshs-koeln.de (L.D.)

**Keywords:** HIIT, Tabata, TRX, RCT, fitness, core, power, functional training

## Abstract

The present study examined the effects of a functional high-intensity suspension training (Functional_HIIT_) on resting blood pressure, psychological well-being as well as on upper body and core strength and cardiorespiratory fitness in moderately trained participants. Twenty healthy, moderately trained adults (10 males and 10 females; age: 36.2 ± 11.1 years, BMI: 23.9 ± 3.7) were randomly assigned to a Functional_HIIT_ training group or passive control group (CON). Functional_HIIT_ performed 16 sessions (2× week for eight weeks, 30 min per session), whereas CON maintained their habitual lifestyle using a physical activity log. Before and after Functional_HIIT_ intervention, resting blood pressure and quality of life (short version of the WHO Quality of Life questionnaire (WHOQOL-BREF)) were assessed. Furthermore, maximum-repetition (leg press, chest press, pulldown, back extension) and trunk muscle strength (Bourban test) as well as cardiorespiratory fitness (Vameval test), were measured before and after the intervention. Both systolic and diastolic blood pressure and WHOQOL-BREF did not change significantly but both showed moderate training-induced effects (0.62 < standardized mean difference (SMD) < 0.82). Significant improvements in the Functional_HIIT_ group were evident on leg press (*p* < 0.01), chest press (*p* < 0.05), and left side Bourban test (*p* < 0.05). Cardiorespiratory fitness did not reveal any time effects or time × group interactions. The present study revealed that eight weeks of Functional_HIIT_ represents a potent stimulus to improve health-related parameters in young adults, whereas Functional_HIIT_ was not sufficient to improve cardiorespiratory fitness.

## 1. Introduction

In the Global Burden of Disease Study 2016, high systolic blood pressure (BP) was mentioned as one of the three leading risk factors for disability-adjusted life years [1]. To address this issue and lower resting BP, physical activity is recommended [2]. While the lowering effect of low-to-moderate intensity aerobic endurance training on resting BP has been evident for a long time [3,4], the positive effect of resistance training on BP remained unclear for a long period of time [5]. Meanwhile, recent meta-analysis demonstrates evidence for lowering effects of resistance training on BP [6,7]. Institutions such as the American Heart Association included resistance training in their guidelines for prevention of cardiovascular diseases [8].

While most of the earlier research predominantly focused on submaximal aerobic exercises, more recent studies have investigated the effect of aerobic high-intensity interval training on BP with positive results [9,10].

In 1996, Tabata and colleges [11] introduced a high-intensity training protocol with a duration of 3–4 minutes, showing similar or even better effects on aerobic and anaerobic endurance parameters than a 60 minute training at moderate intensity. For six weeks, the participants performed 7–8 bouts of 20 second cycling intervals at 170% of VO2_max_ with 10 seconds of rest between sets. Due to difficulties in reproducing the protocol in day-to-day training, different modified Tabata-based protocols were summarized under the title “Tabata-like protocols” [12]. The use of those protocols was found to be effective in well-trained athletes [13,14] in individuals with a sedentary lifestyle [15,16], as well as in top-level athletes [17,18]. Although initially developed in the context of endurance training, the application of “20 seconds loading—10 seconds resting” protocols in strength training also revealed positive effects [19,20]. High-intensity functional training (Functional_HIIT_) is described as a form of exercise including functional, multi-joint movements performed in repeated bouts with high intensities and short intervals of recovery [21,22]. Exercise programs with repeated high-intensity intervals aiming to enhance cardiovascular, metabolic, and psychological health are increasingly applied and analyzed in sport and exercise science [22,23,24,25]. In addition to positive adaptations in sedentary and recreational trained individuals on cardiovascular, metabolic, and psychological levels, researchers advocate that high-intensive interval training requires less time [23,24], since perceived lack of time appears to be a major reason for not exercising [26].

Due to the good time–benefit ratio, Functional_HIIT_ has been found to be a highly effective training method for different populations [22,25]. However, the effectiveness of the Tabata protocol in high-intensity functional suspension training has not been analyzed yet.

Beyond the cardiovascular and metabolic level, an analysis considering the musculoskeletal system in context with Functional_HIIT_ seems reasonable: Low back pain is considered as one of the leading causes of DALYs that do not cause death [1]. Especially in high-income countries there is a high prevalence for low back pain [27]. In a large-scale study, including more than 400,000 adolescents from 28 countries, 37% of participants stated they suffer from low back pain once a month or more frequently [28]. Low back pain is associated with trunk extensor weakness [29], therefore exercise is suggested to be effective for prevention of the latter [30]. Constant exercise, associated with an increase in trunk muscle strength, reduces the risk of back injuries [31]. Higher muscle activity and co-activation of trunk muscles were found in motions such as bending and twisting [32]. It has also been shown that training on unstable surfaces provides higher muscle activation [33]. Therefore, it seems likely that a dynamic exercise, performed on unstable surfaces and with high intensity, increases the muscle activity and co-activity of trunk muscle and potentially contributes to an increase in chest and upper back muscle strength. An increase in chest and upper back muscle strength could potentially reduce the prevalence of low back pain and consequently demonstrate an impact on quality of life.

Therefore, the present study aimed at investigating whether a high-intensity functional suspension training based on the Tabata protocol (Functional_HIIT_) effects resting blood pressure, perceived quality of live, and upper body and core strength. Moreover, we aimed to analyze whether such a high-intensity functional training affects participant’s cardiorespiratory fitness. In the present study, resting blood pressure and perceived quality of live represent the primary outcomes, strength performance the secondary outcome, and cardiorespiratory fitness the tertiary outcome.

## 2. Methods

### 2.1. Study Design and Participants

The present study was conducted as a longitudinal, two-armed, randomized controlled interventional pilot study. Pre- and post-testing were intra-individually performed at a similar time of the day (before noon and after noon). In order to minimize influences of unspecific training loads, both groups were asked to refrain from any changes of habitual physical activity and participants kept a physical activity log. The functional high-intensity suspension training group (Functional_HIIT_) performed additionally to their habitual physical activities a total of 16 instructed and supervised high-intensity functional training sessions (two sessions per week) over a period of eight weeks. The training characteristic and target body regions of interest (exercise type, frequency, volumes, intensity, heart rate, and perceived exercise exertion) were kept similar in each session of Functional_HIIT_.

In contrast to the Functional_HIIT_ group, the control group (CON) merely maintained their weekly training and physical activity regimen of 1.5 ± 0.9 gym training sessions per week.

Twenty healthy, moderately trained adults (10 females and 10 males; age: 36.2 ± 11.1 years; BMI: 23.9 ± 3.7 kg/m^2^) were enrolled in the present randomized controlled training study (Table 1). Participants fulfilled eligibility criteria if they had at least three months of continuous gym experience and were currently training in a gym between 1 and 4 sessions per week. Exclusion criteria were any type of cardiac disease, hypertension, diabetes, use of medication potentially influencing blood pressure, and total endoprosthesis. Participants were asked to refrain from any other activity 24 hours before the exercise sessions.

The study protocol fulfilled the criteria of the Code of Ethics for human experimentation, the Declaration of Helsinki [34], and was approved by the local ethical committee of German Sport University Cologne. All participants received relevant study information, including the potential risks and benefits of the study protocol and signed a written informed consent prior to the start of the study.

An online software (www.randomization.com) was utilized to randomize the participants to the Functional_HIIT_ group, respectively to the control group.

### 2.2. Functional_HIIT_ Training Sessions

Each session of the Functional_HIIT_ group was guided by an experienced instructor. In total, 32 guided Functional_HIIT_ training sessions were offered during the eight-week intervention period (four training sessions per week). Participants were free to choose two training sessions per week, but participants were obliged to attend two training sessions a week during the eight-week intervention period. Overall, each participant performed a total of 16 guided group training sessions. None of the participants had to be excluded due to absence and no participant dropped-out for other reasons.

Mean duration of Functional_HIIT_ sessions was 30 ± 2 minutes and each session consisted of eight different exercises performed with the Tabata protocol. Exercises were mainly performed on a TRX suspension trainer (V-shaped configuration with two independent anchors). To obtain an effect on the cardiorespiratory system, participants performed exercises with whole-body muscle activation (e.g., squats, burpees, jumping jacks, chest press, mountain climbers, squats and rows, stand up/lay down, high knees, push-ups, and crunches; for the full training program please see supplementary file). Ratings of perceived exertion, stress, motivation, and heart rate were documented during Functional_HIIT_ sessions to control for internal load. The CON group maintained their individual, not standardized training. Form of training and duration were recorded in order to trace possible discrepancies between groups.

### 2.3. Testing Procedures

#### 2.3.1. Blood Pressure

At the first visit to the laboratory, blood pressure was measured after five minutes of supervised sitting. Blood pressure was measured manually by an experienced examiner with a mercury column sphygmomanometer (Erkamater™ E300, Germany). All blood pressure measurements were performed by the same examiner with the same device. Blood pressure was measured three times on the dominant arm with a rest interval of 5 min between measurements. The mean of the lowest two values was calculated and included for analyses.

#### 2.3.2. Questionnaires

The WHO Quality of Life questionnaire (WHOQOL-BREF, validated German version) as well as the 10-item version of the Perceived Stress Scale (PSS) were used to measure quality of life and personal well-being. The WHOQOL-BREF consists of 26 questions, which are divided into four sections (environment, physical, psychological, social relationships). Each question is answered by using a five-point Likert scale reaching from “not at all/never” to “extremely/completely/always” [35]. The Perceived Stress Scale developed by Cohen and colleagues [36] and transformed into a 10-item version [37] is widely used to measure the personal perception of stress. The 10-item version is considered to be reliable and valid [38].

#### 2.3.3. Maximum-Repetition Test

In order to determine local muscular endurance, participants performed a maximum-repetition test on four different strength machines. Strength was tested on the leg press (LP) with 150% of the individual’s body weight (BW), the chest press (CP) with 40% BW, pulldown (PD) with 65% BW, and on the back extension machine (BE) with 80% BW. All tests were performed until (1) volitional exhaustion was reached or (2) the predetermined speed of two seconds of each eccentric and concentric work phase could not be maintained during more than two repetitions. Every test was followed by four minutes of passive recovery. One trial for each machine was performed. Before every maximum-repetition test was performed, perceived stress was assessed by using the PSS. After the completion of each test, participants rated their perceived exertion on a scale of 1–10 (CR-10 RPE (rate of perceived exhaustion) scale) [39]. Originally designed for endurance efforts, the use of the CR-10 RPE scale for perceived exertion during strength training is also considered reliable [40].

#### 2.3.4. Trunk Muscle Strength

In addition to maximum-repetition tests, core strength was examined by using the Bourban test. This test was developed by Bourban and colleagues [41] to evaluate trunk muscle strength. It consists of three exercises for the ventral (Bv) and the lateral (left (Bl) and right side (Br)) trunk muscle chain. To examine the ventral trunk muscle chain, participants were asked to take the plank position (ventral position, forearms flat on the ground, toes on the ground) and lift their feet 2–5 cm alternating with fully extended knees at a rate of one elevation per second. Lateral trunk muscle chain was assessed by taking the side plank position (on the side, forearm directly below the shoulder flat on the ground, feet together) and moving the hip toward the floor and back in an extended position (body is in a straight line from the head to the feet) at a rate of two seconds of elevation and two seconds of lowering. According to Tschopp and colleagues [42] the ventral and lateral tests can both be considered as reliable (CV = 14.1%, r = 0.87 and CV = 14.6%, r = 0.81). After each exercise, perceived exertion was assessed by using the RPE scale. One trial for each position was performed.

#### 2.3.5. Cardiorespiratory Fitness

In order to assess cardiorespiratory fitness, participants performed an increasing treadmill test (Vameval test) until volitional exhaustion. During the Vameval test, heart rate was monitored with a heart rate monitor and chest strap (Polar Electro© Oy, Kempele, Finland). The Vameval test is a modified version of the University of Montreal Track Test (UM-Track Test) [43]. The Vameval test is commonly used to determine cardiorespiratory fitness among different groups [44,45,46]. Following the initial phase (200 m at a speed of 8.0 km·h^−1^), velocity increased by 0.5 km·h^−1^ every 200 m until exhaustion. A high correlation between maximal running speed before exhaustion and VO2_max_ determined in laboratory testing is reported for UM-Track Test (r = 0.96) [43]. In the Vameval test, a good test–retest reliability was demonstrated with a coefficient of variation of 3.5% [47].

### 2.4. Statistics

Demographic and performance data are provided as means with standard deviations (SD). All outcome parameters were initially analyzed for normal distribution (Kolmogorov–Smirnov test) and variance homogeneity (Levene test). Separate 2 (group: INT vs. CON) × 2 (time: pre vs. post) repeated measures analyses of variances (rANOVA) were calculated for each outcome measure in order to calculate time × effects as well as time × group interactions. Thereby, baseline values were included as covariate. In case of significant time × group interactions for the respective parameters, the Tukey HSD post hoc tests were additionally performed accompanied by computing standardized mean differences as a measure of pairwise effect size estimation. For pairwise effect size estimation, standardized mean differences (SMD) were also computed (SMD, trivial: d < 0.2, small: 0.2 ≤ d < 0.5, moderate: 0.5 ≤ d < 0.8, large d ≥ 0.8). Additionally, baseline data were checked for significant differences between the two groups, applying Student’s t-test for unpaired samples.

## 3. Results

### 3.1. Baseline Data

All parameters measured at baseline did not differ between the two groups (lowest *p* ≥ 0.213; SMD ≤ 0.31).

### 3.2. Blood Pressure

Both systolic and diastolic blood pressure did not reveal any main or interaction effect (*p* > 0.05) (Table 2). However, moderate effect sizes (SMD: 0.62) were found for Functional_HIIT_ between pre and post testing.

### 3.3. Questionnaires

No significant group × time interaction was found for the entire WHO-QOL (Table 3). Interestingly, standardized mean differences (SMD) of Functional_HIIT_ group for the subdomains regarding psychological health (0.38; 0.09), psychological (0.76; −0.50), social relationships (0.38; 0.00), and environment (0.05; −0.34) revealed partly moderate and relevant changes (Table 3).

### 3.4. Maximum-Repetition Test and Trunk Muscle Strength

A significant group × time interaction was found for maximum-repetition tests on pulldown (*p* = 0.008) and chest press (*p* = 0.016) as well as for the lateral (left) Bourban test (*p* = 0.024) in favor of the Functional_HIIT_ group (Table 4). A significant time effect between pre- and post-test on leg press (37.0 ± 23.82 repetitions vs. 52.1 ± 21.38 repetitions; *p* < 0.01), on chest press (20.6 ± 10.23 repetitions vs. 26.5 ± 11.89 repetitions; *p* < 0.05), and lateral (left) Bourban test (58.22 ± 28.71 s vs. 70.22 ± 24.53 s; *p* < 0.05) was evident.

### 3.5. Cardiorespiratory Fitness

Maximal running velocity in Vameval test did not reveal any main or interaction effect (*p* > 0.05) (Table 5).

## 4. Discussion

The present study aimed at investigating the effect of a suspension training using the “Tabata protocol” on blood pressure, personal well-being, and life quality as well as on trunk strength performance and cardiorespiratory fitness. Systolic and diastolic blood pressure and quality of life were only slightly affected by the Functional_HIIT_ intervention, while considerable increases in trunk strength were revealed. Maximal running velocity in the Vameval test did not change over time, and no interaction effect between the two groups was evident.

In a meta-analysis by Gillison and colleagues [48], the effect of physical exercise on quality of life was assessed. A small but significant positive effect was found in clinical populations after three to six months of exercise. Studies conducted in healthy populations also tend to show positive effects, although only limited data are available in this regard [49]. Recent studies demonstrated that similar interventions with functional high-intensity training evoke improvements in certain dimensions of quality of life in untrained [22] and overweight [25] participants. In a group of healthy adults aged 36–45 years, the WHOQOL-BREF score increased significantly after 13 weeks of moderate-intensity strength and endurance training combined with basic coordination and flexibility exercises [50]. However, health-related quality of life scores have repeatedly been found to be higher in studies working with higher intensities [51,52]. Taking these findings into account, our training intervention consisting of high-intensity exercise in a group-setting was designed to induce improvements of well-being. Nevertheless, in the present study, analysis revealed no significant effects of Functional_HIIT_ on quality of life. This may be due to the fact, that participants were members of a fitness club and physically active before the beginning of the intervention that alteration of the level of physical activity with the present exercise intervention was not sufficient.

Despite the positive adaptations following aerobic high-intensity interval training in sedentary and recreational trained individuals in less training time [23,24], some critical considerations on aerobic HIIT were made. Elevated exertion during and pain following to aerobic HIIT may discourage untrained individuals from performing regular physical activity in the form of HIIT [53,54]. In contrast to those concerns, previous studies demonstrated no negative effect of high-intensity exercise on perceived pain in female participants training with Functional_HIIT_ [22], respectively in female patients performing aerobic HIIT [55]. Nevertheless, future studies should assess whether untrained individuals can train HIIT habitually for longer periods without an increased perception of exertion during or pain following HIIT.

As stated previously, the prevalence of low back pain among adolescents is relatively high [28]. This is of particular interest, because adolescents suffering from low back pain are 3.5 times more likely to be afflicted by low back pain during adulthood [56]. Recent research suggested that exercise is effective for preventing low back pain [30]. Additionally, regular exercise, contributing to an increase in trunk muscle strength, reduces the risk of back injuries [31]. The results of the present study are in line with these findings since the present Functional_HIIT_ intervention revealed improvements in core muscle strength, which represents a protective factor against low back pain and back injuries. It seems evident that the dynamic exercises in the present study, in combination with training on an unstable surface and the high intensities caused an increase in trunk muscle strength. In this regard the improvements in strength in the present study are in line with similar studies demonstrating an increase in functional strength following nine weeks of high-intensity circuit training [25], compared to four weeks of functional high-intensity circuit training [22]. Furthermore, a recent study demonstrated that functional resistance training was sufficient to improve strength and functional strength related performance as well as body pain in females with chronic low-back pain [57]. The present exercise intervention of Functional_HIIT_ consisted of multi-joint exercises performed with high intensities and very little rest between intervals. Therefore, the Functional_HIIT_ was supposed to improve local muscle endurance, muscle strength as well as cardiorespiratory endurance performance. As indicated by the significant effects of Functional_HIIT_ for selected strength tests, the present intervention was sufficient to induce performance improvements in the recreational trained participants. However, it is unclear whether the Functional_HIIT_ training induced adaptions in neuro-muscular structure and/or function as traditional strength training or if the Functional_HIIT_ enhanced local muscular anaerobic endurance. Even though hypertrophic adaptations to eccentric and concentric strength training occurs promptly [58], the time span for increases in muscular protein mass require periods up to months [59]. Therefore, we suggest that the enhancement in functional strength, namely, maximum repetition, in the present study, induced by the 16 Functional_HIIT_ sessions during the eight weeks reflects neural adaptation or increases in local muscular endurance.

The absence of significant changes in maximum strength at back extension may be due to great interindividual differences, which can be seen in the high standard deviation (Table 2).

In contrast to the improvements in strength performance, no improvement in running performance was evident following Functional_HIIT_. A poor cardiorespiratory fitness is considered as a risk factor for cardiovascular diseases [60] and premature death [61]. Although the Functional_HIIT_ exercises consisted predominantly of resistance training exercises, involving large muscle groups (e.g., squats, burpees, jumping jacks, squats and rows, stand up/lay down, high knees, etc.) performed with the own body weight and with high intensities and short recovery periods, endurance performance was unaltered by Functional_HIIT_. We suggest that several factors may explain this result: (i) the exercise mode of Functional_HIIT_ was predominantly related to functional strength, rather than endurance-specific exercise modes (i.e., running- or cycling-based exercise modes), this may not have evoked a sufficient stimulus to improve cardiorespiratory fitness in the participants; (ii) it is possible that the overall intensities (in terms of % of the one-repetition maximum) during Functional_HIIT_ were not high enough to increase cardiorespiratory fitness. A recent meta-analysis suggested for resistance circuit-based training programs a total volume of 14–30 sessions during a period of 6–12 weeks performed with intensities of 60%–90% of the one-repetition maximum for improving VO2_peak_ [62]. Thus, a recent intervention involving similar functional movements, but longer sessions (>60 vs. 30 min) over a slightly longer period (9 vs. 8 weeks) than our Functional_HIIT_ improved VO2_peak_ by ~10% [25]. On the other hand, recent studies applying similar Functional_HIIT_ protocols, with different populations yielded just as little improvements in VO2_max_ [22], especially in endurance running performance [63] as in the present study.

Prolonged elevated blood pressure leads to a reduction of elastin and an increment of collagen and calcium in arterial walls, which results in a reduced elasticity of the artery [64]. The resulting impairments in flexibility in combination with hypertension increase the risk of damage of the arterial wall [65]. Aerobic exercise training has been found to be effective in decreasing arterial stiffness [66], while studies concerning strength training showed a wide range of results, such as no changes [67], decrease [68], or increase [69] of arterial stiffness. In particular, it is commonly concluded that the intensity of strength training affects the outcome of an intervention. In a meta-analysis [70], it was suggested that intense muscle contraction increases intramuscular pressure, leading therefore to high stress in artery walls which results in adaptations increasing arterial stiffness. Arterial stiffness being named as the leading cause for an increase of systolic blood pressure [71], induced some authors to express a critical view on strength training with heavy weights in high-risk populations [72]. While it has been shown that exercise intensity, duration of the exercise and activated muscle mass determine the adaptions of blood pressure during exercise [73], Van Hoof and colleagues [74] mention that in most of the controlled randomized studies available, strength training did not reduce blood pressure. Although a short duration of intervention is sometimes speculated to be a possible explanation for insignificant effects [74], Cornelissen and colleagues [75] did not find a significant relationship between the reduction of blood pressure and the duration of the intervention or the volume of training in their meta-analysis.

However, we did find tendencies that indicate changes in blood pressure, but the altering effect cannot be considered as significant (SMD). This may be because the Functional_HIIT_ was only performed with own body weight. Additional weight could potentially have an important role in blood pressure adjustments. Thus, further research is needed to determine the mechanism by which strength training, especially, Functional_HIIT_, influences blood pressure.

The present study includes some limitations which are worth mentioning. During testing for cardiorespiratory fitness with the Vameval test on the treadmill, we were not able to control for volitional exhaustion with the help of objective parameters such as blood lactate levels or respiratory exchange ratio.

## 5. Conclusions

The aim of the present interventional pilot study was to analyze the effect of a high-intensity functional suspension training based on the “Tabata protocol” (Functional_HIIT_) on upper body as well as core strength, quality of life, resting blood pressure, and endurance performance in moderately subjects. Our data suggest that Functional_HIIT_ has an effect on strength performance in upper body and lower extremities. Effects on systolic blood pressure profiles and certain subdomains of psychological well-being were very limited, since differences were not significant and effect sizes revealed only small-to-moderate effects. Functional_HIIT_ exhibits no effect on endurance performance. Future research should clarify whether Functional_HIIT_ could affect blood pressure and psychological well-being by applying a longer intervention window, higher intensities, and a higher training frequency per week. In those future studies, the varying aspects of strength training, such as complexity, load, time under tension, volume, and duration of a training program should be taken into account.

## Figures and Tables

**Table 1:** Anthropometric characteristics of participants at baseline (Mean ± SD).

	Functional_HIIT_ (n = 10)	CON (n = 10)
Gender [f/m]	5/5	5/5
Age [years]	35 ± 12	37 ± 10
BMI [kg·m^−2^]	22.8 ± 3.4	24.9 ± 4.4

Functional_HIIT_—high-intensity functional training; CON—control. BMI—body mass index.

**Table 2:** Changes in systolic and diastolic blood pressure (mmHg) in Functional_HIIT_ and CON (Mean ± SD).

Test	Functional_HIIT_ Pre	Functional_HIIT_ Post	SMD	CON Pre	CON Post	SMD
Sys bp	131.4 ± 15.3	122.8 ± 12.2	0.62	129.7 ± 17.5	128.5 ± 18.0	0.07
Dia bp	82.7 ± 10.1	77.6 ± 12.5	0.44	82.4 ± 11.4	81.2 ± 12.5	0.10

Sys bp: systolic blood pressure. Dia bp: diastolic blood pressure. No significant changes were found, neither for group × time nor time.

**Table 3:** Changes in quality of life (points) in INT and CON (Mean ± SD).

Test	Functional_HIIT_ Pre	Functional_HIIT_ Post	SMD	CON Pre	CON Post	SMD
Physical Health	22.2 ± 2.0	22.8 ± 1.2	0.38	23.6 ± 2.5	23.8 ± 2.1	0.09
Psychological	22.1 ± 2.6	23.8 ± 1.9	0.76	21.6 ± 2.0	20.8 ± 1.2	−0.50
Social Relationships	11.8 ± 1.4	12.4 ± 1.8	0.38	12.7 ± 2.2	12.7 ± 1.7	0.00
Environment	32.8 ± 1.5	32.9 ± 2.9	0.05	32.7 ± 2.7	31.8 ± 2.6	−0.34

No significant changes were found, neither for group × time nor for time.

**Table 4:** Changes in strength parameters measured with leg press (LP; repetitions), chest press (CP; repetitions), pulldown (PD, repetitions), back extension (BE, repetitions), Bourban test ventral side (B_v_, seconds), Bourban test lateral left side (B_l_), Bourban test lateral right side (B_r_), in Functional_HIIT_ and CON (Mean ± SD).

Test	Functional_HIIT_ Pre	Functional_HIIT_ Post	SMD	CON Pre	CON Post	SMD	Time	Time × Group
LP	37.0 ± 23.8	52.1 ± 21.4	0.67	39.6 ± 20.0	46.4 ± 32.3	0.26	*p* = 0.008 *	*p* = 0.278
CP	20.6 ± 10.2	26.5 ± 11.9 *	0.53	34.2 ± 18.9	34.0 ± 18.5	−0.01	*p* = 0.024 *	*p* = 0.016 *
PD	23.3 ± 14.2	36.8 ± 22.3 **	0.74	25.8 ± 13.0	23.3 ± 12.4	−0.19	*p* = 0.053	*p* = 0.008 *
BE	45.9 ± 33.1	67.9 ± 59.2	0.48	41.6 ± 13.2	40.1 ± 13.3	−0.11	*p* = 0.153	*p* = 0.106
B_v_	108.1 ± 75.1	121.0 ± 67.3	0.18	150.2 ± 53.5	152.3 ± 57.5	0.03	*p* = 0.749	*p* = 0.892
B_l_	58.2 ± 28.7	70.2 ± 24.5 **	0.45	65.4 ± 17.3	65.8 ± 16.6	0.02	*p* = 0.018 *	*p* = 0.024 *
B_r_	58.2 ± 25.7	67.0 ± 26.7	0.34	72.2 ± 19.6	70.6 ± 24.4	−0.07	*p* = 0.610	*p* = 0.398

*: significant effect; LP: leg press; CP: chest press; PD: pulldown; BE: back extension. B_v_: trunk strength of ventral side in the Bourban test, in seconds; B_l_: trunk strength of lateral left side in the Bourban test, in seconds; B_r_: trunk strength of lateral right side in the Bourban test, in seconds.

**Table 5:** Changes in maximal running velocity (km/h^−1^), heart rate (beats per minute), and rates of perceived exertion (arbitrary units) in Vameval test in Functional_HIIT_ and CON (Mean ± SD).

Parameter	Functional_HIIT_ Pre	Functional_HIIT_ Post	CON Pre	CON Post	Time	Time × Group
V_max_	14.0 ± 2.5	14.5 ± 2.2	15.6 ± 2.2	15.6 ± 2.2	0.189	0.189
HR_max_	193.1 ± 6.7	190.8 ± 6.8	189.2 ± 7.1	190.3 ± 7.5	0.379	0.158
RPE	9.8 ± 0.4	10.0 ± 0.0	9.7 ± 0.7	9.0 ± 1.5	0.463	0.295

V_max_: running velocity in the last completed stage of Vameval test. HR_max_: mean heart rate during last completed stage of Vameval test; RPE: rates of perceived exertion in the last completed stage of Vameval test.

## Supplementary Files

Supplementary File 1

## References

[1] Global Risk Factors Collaboraters (2017). Global, regional, and national comparative risk assessment of 84 behavioural, environmental and occupational, and metabolic risks or clusters of risks, 1990–2016: A systematic analysis for the Global Burden of Disease Study 2016. Lancet.

[2] US Departement of Health and Human Services (1996). Physical Activity and Health: A Report of the Surgeon General.

[3] Hartley L.H., Grimby G., Kilbom Å., Nilsson N.J., Åstrand I., Bjure J., Ekblom B., Saltin B. (1969). Physical Training in Sedentary Middle-aged and Older Men III. Cardiac Output and Gas Exchange at Submaximal and Maximal Exercise. Scand. J. Clin. Lab. Investig..

[4] Choquette G., Ferguson R.J. (1973). Blood pressure reduction in “borderline” hypertensives following physical training. CMA J..

[5] Kelley G.A., Kelley K.S. (2000). Progressive Resistance Exercise and Resting Blood Pressure. A Meta-Analysis of Randomized Controlled Trials. Hypertension.

[6] Figueroa A., Okamoto T., Jaime S.J., Fahs C.A. (2019). Impact of high- and low-intensity resistance training on arterial stiffness and blood pressure in adults across the lifespan: A review. Pflügers Arch. Eur. J. Physiol..

[7] Inder J.D., Carlson D.J., Dieberg G., McFarlane J.R., Hess N.C., Smart N.A. (2016). Isometric exercise training for blood pressure management: A systematic review and meta-analysis to optimize benefit. Hypertens. Res..

[8] Brook R.D., Appel L.J., Rubenfire M., Ogedegbe G., Bisognano J.D., Elliott W.J., Fuchs F.D., Hughes J.W., Lackland D.T., Staffileno B.A. (2013). Beyond medications and diet: Alternative approaches to lowering blood pressure: A scientific statement from the american heart association. Hypertension.

[9] Molmen-Hansen H.E., Stolen T., Tjonna A.E., Aamot I.L., Ekeberg I.S., Tyldum G.A., Wisloff U., Ingul C.B., Stoylen A. (2011). Aerobic interval training reduces blood pressure and improves myocardial function in hypertensive patient. Eur. J. Prev. Cardiol..

[10] Grace F., Herbert P., Elliott A.D., Richards J., Beaumont A., Sculthorpe N.F. (2018). High intensity interval training (HIIT) improves resting blood pressure, metabolic (MET) capacity and heart rate reserve without compromising cardiac function in sedentary aging men. Exp. Gerontol..

[11] Tabata I., Nishimura K., Kouzaki M., Hirai Y., Ogita F., Miyachi M., Yamamoto K. (1996). Effects of moderate-intensity endurance and high-intensity intermittent training on anaerobic capacity and VO2max. Med. Sci. Sports Exerc..

[12] Viana R.B., De Lira C.A., Naves J.P., Coswig V.S., Del Vecchio F.B., Gentil P. (2018). Tabata protocol: A review its application, variations and outcomes. Clin. Physiol. Funct. Imaging.

[13] Ravier G., Dugué B., Grappe F., Rouillion J.D. (2009). Impressive anaerobic adaptations in elite karate athletes due to few intensive intermittent sessions added to regular karate training. Scand. J. Med. Sci. Sports.

[14] Invernizzi P.L., Longo S., Scurati R., Maggioni M.A., Michielon G. (2014). Interpretation and perception of slow, moderate, and fast swimming paces in distance and sprint swimmers. Percept. Mot. Skills.

[15] Jabbour G., Iancu H.-D., Paulin A. (2015). Effects of High-Intensity Training on Anaerobic and Aerobic Contributions to Total Energy Release During Repeated Supramaximal Exercise in Obese Adults. Sports Med. Open.

[16] Joanisse S., McKAy B.R., Nederveen J.P., Scribbans T.D., Gurd B.J., Gillen J.B., Gibala M.J., Tarnopolsky M., Parise G. (2015). Satellite cell activity, without expansion, after nonhypertrophic stimuli. Am. J. Physiol. Regul. Integr. Comp. Physiol..

[17] Vourimaa T., Vasankari T., Rusko H. (2000). Comparison of Physiological Strain and Muscular Performance of Athletes during Two Intermittent Running Exercises at the Velocity Associated with VO2max. Int. J. Sports Med..

[18] Nicolò A., Bazzucchi I., Lenti M., Haxhi J., di Palumbo A.S., Sacchetti M. (2014). Neuromuscular and metabolic responses to high-intensity intermittent cycling protocols with different work-to-rest ratios. Int. J. Sports Physiol. Perform..

[19] Fortner H.A., Salgado J.M., Holmstrup A.M., Holmstrup M.E. (2014). Cardiovascular and Metabolic Demands of the Kettlebell Swing using Tabata Interval versus a Traditional Resistance Protocol. Int. J. Exerc. Sci..

[20] Williams B.M., Kraemer R.R. (2015). Comparison of cardiorespiratory and metabolic responses in kettlebell high-intensity interval training versus sprint interval cycling. J. Strength Cond. Res..

[21] Feito Y., Heinrich K.M., Butcher S.J., Posten W.S. (2018). High-Intensity Functional Training (HIFT): Definition and Research Implications for Improved Fitness. Sports.

[22] Sperlich B., Hahn L.S., Edel A., Behr T., Helmprobst J., Leppich R., Wallmann-Sperlich B., Holmberg H.-C. (2018). A 4-Week Intervention Involving Mobile-Based Daily 6-Minute Micro-Sessions of Functional High-Intensity Circuit Training Improves Strength and Quality of Life, but Not Cardio-Respiratory Fitness of Young Untrained Adults. Front. Physiol..

[23] Kessler H.S., Sisson S.B., Short K.R. (2012). The potential for high-intensity interval training to reduce cardiometabolic disease risk. Sports Med..

[24] Milanović Z., Sporiš G., Weston M. (2015). Effectiveness of High-Intensity Interval Training (HIT) and continuous endurance training for VO2max Improvements: A systematic review and meta-analysis of controlled trials. Sports Med..

[25] Sperlich B., Wallmann-Sperlich B., Zinner C., Von Stauffenberg V., Losert H., Holmberg H.-C. (2017). Functional High-Intensity Circuit Training Improves Body Composition, Peak Oxygen Uptake, Strength, and Alters Certain Dimensions of Quality of Life in Overweight Women. Front. Physiol..

[26] Godin G., Desharnais R., Valois P., Lepage L., Jobin J., Bradet R. (1994). Differences in perceived barriers to exercise between high and low intenders: Observations among different populations. Am. J. Health Promot..

[27] Maher C., Underwood M., Buchbinder R. (2017). Non-specific low back pain. Lancet.

[28] Swain M.S., Henschke N., Kamper S.J., Gobina I., Ottová-Jordan V., Maher C.G. (2014). An international survey of pain in adolescents. BMC Public Health.

[29] Cho K.H., Beom J.W., Lee T.S., Lim J.H., Lee T.H., Yuk J.H. (2014). Trunk muscle strength as a risk factor nonspecific low back pain: A pilot study. Ann. Rehabil. Med..

[30] Steffens D., Maher C.G., Pereira L.S., Stevens M.L., Oliveira V.C., Chapple M., Teixeira-Salmela L.F., Hancock M.J. (2016). Prevention of Low Back Pain. A Systematic Review and Meta-analysis. JAMA Intern. Med..

[31] Peate W.F., Bates G., Lund K., Francis S., Bellamy K. (2007). Core strength: A new model for injury prediction and prevention. J. Occup. Med. Toxicol..

[32] Marras W.S., Mirka G.A. (1992). A Comprehensive Evaluation of Trunk Response to Asymmetric Trunk Motion. Spine.

[33] Marshall P.W., Murphey B.A. (2006). Increased deltoid and abdominal muscle activity during swiss ball bench press. J. Strength Cond. Res..

[34] World Medical Association (2013). Declaration of Helsinki: Ethical principles for medical research involving human subjects. JAMA.

[35] The WHOQOL Group (1998). The World Health Organization Quality of Life Assessment (WHOQOL): Development and general psychometric properties. Soc. Sci. Med..

[36] Cohen S., Kamarack T., Mermelstein R. (1983). A Global Measure of Perceived Stress. J. Health Soc. Behav..

[37] Cohen S., Williamson G., Spacapan S., Oskamp S. (1988). Perceived Stress in a Probability Sample of the United States. The Social Psychology of Health: Claremont Symposium on Applied Social Psychology.

[38] Roberti J.W., Harrington L.N., Storch E.A. (2006). Further Psychometric Support for the 10-Item Version of the Perceived Stress Scale. J. Coll. Couns..

[39] Borg G.A. (1982). Psychophysical bases of perceived exertion. Med. Sci. Sports Exerc..

[40] Day M.L., McGuigan R., Brice G., Foster C. (2004). Monitoring work intensities during resistance training using a session RPE scale. J. Strength Cond. Res..

[41] Bourban P., Hübner K., Tschopp M., Marti B. (2001). Basic requirements of trunk muscle strength in elite sport: Results of a set of 3 standardized tests. Schweiz. Z. Sportmed. Sporttraum..

[42] Tschopp M., Bourban P., Hübner K., Marti B. (2001). Reliability of a standardized, dynamic trunk muscle strength test: Experiences with healthy male elite athletes. Schweiz. Z. Sportmed. Sporttraum..

[43] Léger L., Boucher R. (1980). An Indirect Continuous Running Multistage Field Test. The Université de Montréal Track Test. Can. J. Appl. Sport. Sci..

[44] Buchheit M., Mendez-Villanueva A., Simpson B.M., Bourdon P.C. (2010). Match Running Performance and Fitness in Youth Soccer. Int. J. Sports Med..

[45] Dellal A., Varliette C., Owen A., Chirico E.N., Pialoux V. (2012). Small-sided games versus interval training in amateur soccer players: Effects on the aerobic capacity and the ability to perform intermittent exercises with changes of direction. J. Strength Cond. Res..

[46] Zghal F., Martin V., Thorkani A., Arnal P.J., Tabka Z., Cottin F. (2014). Effects of endurance training on the maximal voluntary activation level of the knee extensor muscles. Eur. J. Appl. Physiol..

[47] Buchheit M., Mendez-Villanueva A. (2013). Reliability and stability of anthropometric and performance measures in highly-trained young soccer players: Effect of age and maturation. J. Sports Sci..

[48] Gillison F.B., Skevington S.M., Sato A., Standage M., Evangelidou S. (2009). The effects of exercise interventions on quality of life in clinical and healthy populations; a meta-analysis. Soc. Sci. Med..

[49] Bize R., Johnson J.A., Plotnikoff R.C. (2007). Physical activity level and health-related quality of life in the general adult population: A systematic review. Prev. Med..

[50] Brand R., Schlicht W., Grossmann K., Duhnsen R. (2006). Effects of a physical exercise intervention on employees’ perceptions of quality of life: A randomized controlled trial. Soz. Praventiv. Med..

[51] Katula J.A., Rejeski W.J., Marsh A.P. (2008). Enhancing quality of life in older adults: A comparison of muscular strength and power training. Health Qual. Life Outcome.

[52] McGrath J.A., O’Malley M., Hendrix T.J. (2011). Group exercise mode and health-related quality of life among healthy adults. J. Adv. Nurs..

[53] Hardcastle S.J., Ray H., Beale L., Hagger M.S. (2014). Why sprint interval training is inappropriate for a largely sedentary population. Front. Psychol..

[54] Biddle S.J.H., Batterham A.M. (2015). High-intensity interval exercise training for public health: A big HIT or shall we HIT it on the head?. Int. J. Behav. Nutr. Phys. Act..

[55] Schmitt J., Lindner N., Reuss-Borst M., Holmberg H.-C., Sperlich B. (2016). A 3-week multimodal intervention involving high-intensity interval training in female cancer survivors: A randomized controlled trial. Physiol. Rep..

[56] Hestbaek L., Leboeuf-Yde C., Kyvik K.O. (2006). Is comorbidity in adolescence a predictor for adult low back pain? A prospective study of a young population. BMC Musculoskelet. Disord..

[57] Cortell-Tormo J.M., Sánchez P.T., Chulvi-Medrano I., Tortosa-Martínez J., Manchado-López C., Llana-Belloch S., Pérez-Soriano P. (2018). Effects of functional resistance training on fitness and quality of life in females with chronic nonspecific low-back pain. J. Back Musculoskelet. Rehabil..

[58] Franchi M.V., Reeves N.D., Narici M.V. (2017). Skeletal muscle remodeling in response to eccentric vs. concentric loading: Morphological, molecular, and metabolic adaptations. Front. Physiol..

[59] Zinner C., Baessler B., Weiss K., Ruf J., Michels G., Holmberg H.C., Sperlich B. (2017). Effect of resistance training with vibration and compression on the formation of muscle and bone. Muscle Nerve.

[60] Blair S.N., Kohl H.W., Paffenbarger R.S., Clark D.G., Cooper K.H., Gibbons L.W. (1989). Physical Fitness and All-Cause Mortality: A Prospective Study of Healthy Men and Women. JAMA.

[61] Bouchard C., Blair S.N., Katzmarzyk P.T. (2015). Less sitting, more physical activity, or higher fitness. Mayo Clin. Proc..

[62] Munoz-Martinez F.A., Rubio-Arias J.A., Ramos-Campo D.J., Alcaraz P.E. (2017). Effectiveness of resistance circuit-based training for maximum oxygen uptake and upper-body one-repetition maximum improvements: A systematic review and meta-analysis. Sports Med..

[63] Engel F.A., Wagner M.O., Schelhorn F., Deubert F., Leutzsch S., Stolz A., Sperlich B. (2019). Classroom-Based Micro-Sessions of Functional High-Intensity Circuit Training Enhances Functional Strength but Not Cardiorespiratory Fitness in School Children—A Feasibility Study. Front. Public Health.

[64] London G.M., Guerin A.P. (1999). Influence of arterial pulse and reflected waves on blood pressure and cardiac function. Am. Heart J..

[65] Laurent S., Cockcroft J., Van Bortel L., Boutouyrie P., Giannattasio C., Hayoz D., Pannier B., Vlachopoulos C., Wilkinson I., Struijker-Boudier H. (2006). On behalf of the European Network for non-invasive investigation of large arteries. Expert consensus document on arterial stiffness: Methodological issues and clinical applications. Eur. Heart J..

[66] Fournier S.B., Donley D.A., Bonner D.E., Devallance E., Olfert I.M., Chantler P.D. (2015). Improved Arterial-Ventricular Coupling in Metabolic Syndrome after Exercise Training: A Pilot Study. Med. Sci. Sports Exerc..

[67] Casey D.P., Beck D.T., Braith R.W. (2007). Progressive resistance training without volume increases does not alter arterial stiffness and aortic wave reflection. Exp. Biol. Med..

[68] Beck D.T., Martin J.S., Casey D.P., Braith R.W. (2013). Exercise training reduces peripheral arterial stiffness and myocardial oxygen demand in young prehypertensive subjects. Am. J. Hypterens..

[69] Miyachi M., Donato A.J., Yamamoto K., Takashi K., Gates P.E., Moreau K.L., Tanaka H. (2003). Greater age-related reductions in central arterial compliance in resistance-trained men. Hypertension.

[70] Montero D., Roberts C.K., Vinet A. (2014). Effect of Aerobic Exercise Training on Arterial Stiffness in Obese Populations. A Systematic Review and Meta-Analysis. Sports Med..

[71] O’Rourke M. (1990). Arterial stiffness, systolic blood pressure, and logical treatment of arterial hypertension. Hypertension.

[72] Miyachi M., Kawano H., Sugawara J., Takahashi K., Hayashi K., Yamazaki K., Tabata I., Tanaka H. (2004). Unfavorable Effects of Resistance Training on Central Arterial Compliance. A Randomized Intervention Study. Circulation.

[73] Lewis S.F., Snell P.G., Taylor W.F., Hamra M., Graham R.M., Pettinger W.A., Blomqvist C.G. (1985). Role of muscle mass and mode of contraction in circulatory responses to exercise. J. Appl. Physiol..

[74] Van Hoof R., Macor F., Linjnen P., Staessen J., Thijs L., Vanhees L., Fagard R. (1996). Effect of Strength Training on Blood Pressure Measured in Various Conditions in Sedentary Men. Int. J. Sports Med..

[75] Cornelissen V.A., Fagard R.H. (2005). Effect of resistance training on resting blood pressure: A meta-analysis of randomized controlled trials. J. Hypertens..

