# Supplementary material for: Can High-Intensity Functional Suspension Training over Eight Weeks Improve Resting Blood Pressure and Quality of Life in Young Adults? A Randomized Controlled Trial
Source: Int J Environ Res Public Health. 2019 Dec 12;16(24):5062. doi: 10.3390/ijerph16245062 (PMC6950016; doi:10.3390/ijerph16245062)
Supplement: Supplementary File 1 [file ijerph-16-05062-s001.pdf]

Details of the FunctionalHIIT training sessions during the 8-week FunctionalHIIT training intervention. Four group training sessions per week were scheduled and offered. Participants had to attend two out of four training sessions per week. Duration of each session was ≈30 minutes. Each exercise was performed with the "Tabata interval protocol 20 seconds loading – 10 seconds resting".

Week	Session	FunctionalHIIT
1	1	3 min warm-up<br>- 8 sets of<br>- 20-s TRX squats + 10-s recovery<br>- 8 sets of<br>- 20-s burpees + 10-s recovery<br>- 8 sets of<br>- 20-s jumping jack + 10-s recovery<br>- 8 sets of<br>- 20-s crunches + 10-s recovery<br>- 8 sets of<br>- 20-s dips + 10-s recovery<br>- 8 sets of<br>- 20-s TRX squats + 10-s recovery<br>3 min cool-down
	2	3 min warm-up<br>- 8 sets of<br>- 20-s jumping jack + 10-s recovery<br>- 8 sets of<br>- 20-s TRX chest press + 10-s recovery<br>- 8 sets of<br>- 20-s mountain climbers + 10-s recovery<br>- 8 sets of<br>- 20-s crunches + 10-s recovery<br>- 8 sets of<br>- 20-s stand up, lay down + 10-s recovery<br>- 8 sets of<br>- 20-s dips + 10-s recovery<br>3 min cool-down
	3	3 min warm-up<br>- 8 sets of<br>- 20-s jumping jack + 10-s recovery<br>- 8 sets of<br>- 20-s TRX chest press + 10-s recovery<br>- 8 sets of<br>- 20-s skippings + 10-s recovery<br>- 8 sets of<br>- 20-s high knees + 10-s recovery<br>- 8 sets of<br>- 20-s mountain climbers + 10-s recovery<br>- 8 sets of<br>- 20-s TRX chest press + 10-s recovery<br>3 min cool-down
	4	3 min warm-up<br>- 8 sets of<br>- 20-s jumping jack + 10-s recovery<br>- 8 sets of

		- 20-s TRX chest press + 10-s recovery<br>- 8 sets of<br>- 20-s crunches + 10-s recovery<br>- 8 sets of<br>- 20-s push-ups + 10-s recovery<br>- 8 sets of<br>- 20-s jumping jack + 10-s recovery<br>- 8 sets of<br>- 20-s lunges + 15-s recovery<br>3 min cool-down
	1	3 min warm-up<br>- 8 sets of<br>- 20-s jumping jack + 10-s recovery<br>- 8 sets of<br>- 20-s burpees + 10-s recovery<br>- 8 sets of<br>- 20-s mountain climbers + 10-s recovery<br>- 8 sets of<br>- 20-s lunges + 10-s recovery<br>- 8 sets of<br>- 20-s high knees + 10-s recovery<br>- 8 sets of<br>- 20-s crunches + 10-s recovery<br>3 min cool-down
2	2	3 min warm-up<br>- 8 sets of<br>- 20-s jumping jack + 10-s recovery<br>- 8 sets of<br>- 20-s TRX chest press + 10-s recovery<br>- 8 sets of<br>- 20-s stand up, lay down + 10-s recovery<br>- 8 sets of<br>- TRX squats and row + 10-s recovery<br>- 8 sets of<br>- 20-s high knees + 10-s recovery<br>- 8 sets of<br>- 20-s push-ups + 10-s recovery<br>3 min cool-down
	3	3 min warm-up<br>- 8 sets of<br>- 20-s TRX chest press + 10-s recovery<br>- 8 sets of<br>- 20-s TRX squats + 10-s recovery<br>- 8 sets of<br>- 20-s mountain climbers + 10-s recovery<br>- 8 sets of<br>- 20-s skippings + 10-s recovery<br>- 8 sets of<br>- 20-s crunches + 10-s recovery<br>- 8 sets of<br>- 20-s stand up, lay down + 10-s recovery<br>3 min cool-down
	4	3 min warm-up<br>- 8 sets of<br>- 20-s mountain climbers + 10-s recovery

		- 8 sets of<br>- 20-s TRX chest press + 10-s recovery<br>- 8 sets of<br>- 20-s skippings + 10-s recovery<br>- 8 sets of<br>- 20-s burpees + 10-s recovery<br>- 8 sets of<br>- 20-s crunches + 10-s recovery<br>- 8 sets of<br>- 20-s TRX squats + 10-s recovery<br>3 min cool-down
3	1	3 min warm-up<br>- 8 sets of<br>- 20-s high knees + 10-s recovery<br>- 8 sets of<br>- 20-s TRX chest press + 10-s recovery<br>- 8 sets of<br>- 20-s TRX squats + 10-s recovery<br>- 8 sets of<br>- 20-s crunches + 10-s recovery<br>- 8 sets of<br>- 20-s stand up, lay down + 10-s recovery<br>- 8 sets of<br>- 20-s push-ups + 10-s recovery<br>3 min cool-down
	2	3 min warm-up<br>- 8 sets of<br>- 20-s jumping jack + 10-s recovery<br>- 8 sets of<br>- 20-s TRX chest press + 10-s recovery<br>- 8 sets of<br>- 20-s stand up, lay down + 10-s recovery<br>- 8 sets of<br>- TRX squats and row + 10-s recovery<br>- 8 sets of<br>- 20-s high knees + 10-s recovery<br>- 8 sets of<br>- 20-s push-ups + 10-s recovery<br>3 min cool-down
	3	3 min warm-up<br>- 8 sets of<br>- 20-s TRX squats + 10-s recovery<br>- 8 sets of<br>- 20-s burpees + 10-s recovery<br>- 8 sets of<br>- 20-s jumping jack + 10-s recovery<br>- 8 sets of<br>- 20-s crunches + 10-s recovery<br>- 8 sets of<br>- 20-s high knees + 10-s recovery<br>- 8 sets of<br>- 20-s TRX squats + 10-s recovery<br>3 min cool-down
	4	3 min warm-up<br>- 8 sets of

		- 20-s jumping jack + 10-s recovery<br>- 8 sets of<br>- 20-s burpees + 10-s recovery<br>- 8 sets of<br>- 20-s mountain climbers + 10-s recovery<br>- 8 sets of<br>- 20-s lunges + 10-s recovery<br>- 8 sets of<br>- 20-s high knees + 10-s recovery<br>- 8 sets of<br>- 20-s crunches + 10-s recovery<br>3 min cool-down
4	1	3 min warm-up<br>- 8 sets of<br>- 20-s TRX squats + 10-s recovery<br>- 8 sets of<br>- 20-s burpees + 10-s recovery<br>- 8 sets of<br>- 20-s stand up, lay down + 10-s recovery<br>- 8 sets of<br>- 20-s lunges + 10-s recovery<br>- 8 sets of<br>- 20-s high knees + 10-s recovery<br>- 8 sets of<br>- 20-s TRX squats and row + 10-s recovery<br>3 min cool-down
	2	3 min warm-up<br>- 8 sets of<br>- 20-s jumping jack + 10-s recovery<br>- 8 sets of<br>- 20-s TRX chest press + 10-s recovery<br>- 8 sets of<br>- 20-s stand up, lay down + 10-s recovery<br>- 8 sets of<br>- TRX squats and row + 10-s recovery<br>- 8 sets of<br>- 20-s high knees + 10-s recovery<br>- 8 sets of<br>- 20-s push-ups + 10-s recovery<br>3 min cool-down
	3	3 min warm-up<br>- 8 sets of<br>- 20-s jumping jack + 10-s recovery<br>- 8 sets of<br>- 20-s burpees + 10-s recovery<br>- 8 sets of<br>- 20-s stand up, lay down + 10-s recovery<br>- 8 sets of<br>- crunches + 10-s recovery<br>- 8 sets of<br>- 20-s push-ups + 10-s recovery<br>- 8 sets of<br>- 20-s lunges + 10-s recovery<br>3 min cool-down
	4	3 min warm-up

		- 8 sets of<br>- 20-s mountain climbers + 10-s recovery<br>- 8 sets of<br>- 20-s TRX chest press + 10-s recovery<br>- 8 sets of<br>- 20-s skippings + 10-s recovery<br>- 8 sets of<br>- 20-s push-ups + 10-s recovery<br>- 8 sets of<br>- 20-s high knees + 10-s recovery<br>- 8 sets of<br>- 20-s TRX squats + 10-s recovery<br>3 min cool-dow
	1	3 min warm-up<br>- 8 sets of<br>- 20-s jumping jack + 10-s recovery<br>- 8 sets of<br>- 20-s burpees + 10-s recovery<br>- 8 sets of<br>- 20-s mountain climbers + 10-s recovery<br>- 8 sets of<br>- 20-s lunges + 10-s recovery<br>- 8 sets of<br>- 20-s high knees + 10-s recovery<br>- 8 sets of<br>- 20-s crunches + 10-s recovery<br>3 min cool-down
5	2	3 min warm-up<br>- 8 sets of<br>- 20-s jumping jack + 10-s recovery<br>- 8 sets of<br>- 20-s TRX chest press + 10-s recovery<br>- 8 sets of<br>- 20-s stand up, lay down + 10-s recovery<br>- 8 sets of<br>- TRX squats and row + 10-s recovery<br>- 8 sets of<br>- 20-s high knees + 10-s recovery<br>- 8 sets of<br>- 20-s push-ups + 10-s recovery<br>3 min cool-down
	3	3 min warm-up<br>- 8 sets of<br>- 20-s TRX chest press + 10-s recovery<br>- 8 sets of<br>- 20-s TRX squats + 10-s recovery<br>- 8 sets of<br>- 20-s mountain climbers + 10-s recovery<br>- 8 sets of<br>- 20-s skippings + 10-s recovery<br>- 8 sets of<br>- 20-s crunches + 10-s recovery<br>- 8 sets of<br>- 20-s stand up, lay down + 10-s recovery<br>3 min cool-down

	4	3 min warm-up<br>- 8 sets of<br>- 20-s mountain climbers + 10-s recovery<br>- 8 sets of<br>- 20-s TRX chest press + 10-s recovery<br>- 8 sets of<br>- 20-s skippings + 10-s recovery<br>- 8 sets of<br>- 20-s burpees + 10-s recovery<br>- 8 sets of<br>- 20-s crunches + 10-s recovery<br>- 8 sets of<br>- 20-s TRX squats + 10-s recovery<br>3 min cool-down
	1	3 min warm-up<br>- 8 sets of<br>- 20-s TRX squats + 10-s recovery<br>- 8 sets of<br>- 20-s burpees + 10-s recovery<br>- 8 sets of<br>- 20-s stand up, lay down + 10-s recovery<br>- 8 sets of<br>- 20-s lunges + 10-s recovery<br>- 8 sets of<br>- 20-s high knees + 10-s recovery<br>- 8 sets of<br>- 20-s TRX squats and row + 10-s recovery<br>3 min cool-down
6	2	3 min warm-up<br>- 8 sets of<br>- 20-s jumping jack + 10-s recovery<br>- 8 sets of<br>- 20-s TRX chest press + 10-s recovery<br>- 8 sets of<br>- 20-s stand up, lay down + 10-s recovery<br>- 8 sets of<br>- TRX squats and row + 10-s recovery<br>- 8 sets of<br>- 20-s high knees + 10-s recovery<br>- 8 sets of<br>- 20-s push-ups + 10-s recovery<br>3 min cool-down
	3	3 min warm-up<br>- 8 sets of<br>- 20-s jumping jack + 10-s recovery<br>- 8 sets of<br>- 20-s burpees + 10-s recovery<br>- 8 sets of<br>- 20-s stand up, lay down + 10-s recovery<br>- 8 sets of<br>- crunches + 10-s recovery<br>- 8 sets of<br>- 20-s push-ups + 10-s recovery<br>- 8 sets of<br>- 20-s lunges + 10-s recovery

		3 min cool-down
	4	3 min warm-up<br>- 8 sets of<br>- 20-s mountain climbers + 10-s recovery<br>- 8 sets of<br>- 20-s TRX chest press + 10-s recovery<br>- 8 sets of<br>- 20-s skippings + 10-s recovery<br>- 8 sets of<br>- 20-s push-ups + 10-s recovery<br>- 8 sets of<br>- 20-s high knees + 10-s recovery<br>- 8 sets of<br>- 20-s TRX squats + 10-s recovery<br>3 min cool-dow
7	1	3 min warm-up<br>- 8 sets of<br>- 20-s TRX squats + 10-s recovery<br>- 8 sets of<br>- 20-s burpees + 10-s recovery<br>- 8 sets of<br>- 20-s jumping jack + 10-s recovery<br>- 8 sets of<br>- 20-s crunches + 10-s recovery<br>- 8 sets of<br>- 20-s dips + 10-s recovery<br>- 8 sets of<br>- 20-s TRX squats + 10-s recovery<br>3 min cool-down
	2	3 min warm-up<br>- 8 sets of<br>- 20-s jumping jack + 10-s recovery<br>- 8 sets of<br>- 20-s TRX chest press + 10-s recovery<br>- 8 sets of<br>- 20-s mountain climbers + 10-s recovery<br>- 8 sets of<br>- 20-s crunches + 10-s recovery<br>- 8 sets of<br>- 20-s stand up, lay down + 10-s recovery<br>- 8 sets of<br>- 20-s dips + 10-s recovery<br>3 min cool-down
	3	3 min warm-up<br>- 8 sets of<br>- 20-s jumping jack + 10-s recovery<br>- 8 sets of<br>- 20-s TRX chest press + 10-s recovery<br>- 8 sets of<br>- 20-s skippings + 10-s recovery<br>- 8 sets of<br>- 20-s high knees + 10-s recovery<br>- 8 sets of<br>- 20-s mountain climbers + 10-s recovery<br>- 8 sets of

		- 20-s TRX chest press + 10-s recovery<br>3 min cool-down
	4	3 min warm-up<br>- 8 sets of<br>- 20-s jumping jack + 10-s recovery<br>- 8 sets of<br>- 20-s TRX chest press + 10-s recovery<br>- 8 sets of<br>- 20-s crunches + 10-s recovery<br>- 8 sets of<br>- 20-s push-ups + 10-s recovery<br>- 8 sets of<br>- 20-s jumping jack + 10-s recovery<br>- 8 sets of<br>- 20-s lunges + 15-s recovery<br>3 min cool-down
8	1	3 min warm-up<br>- 8 sets of<br>- 20-s jumping jack + 10-s recovery<br>- 8 sets of<br>- 20-s burpees + 10-s recovery<br>- 8 sets of<br>- 20-s mountain climbers + 10-s recovery<br>- 8 sets of<br>- 20-s lunges + 10-s recovery<br>- 8 sets of<br>- 20-s high knees + 10-s recovery<br>- 8 sets of<br>- 20-s crunches + 10-s recovery<br>3 min cool-down
	2	3 min warm-up<br>- 8 sets of<br>- 20-s jumping jack + 10-s recovery<br>- 8 sets of<br>- 20-s TRX chest press + 10-s recovery<br>- 8 sets of<br>- 20-s stand up, lay down + 10-s recovery<br>- 8 sets of<br>- TRX squats and row + 10-s recovery<br>- 8 sets of<br>- 20-s high knees + 10-s recovery<br>- 8 sets of<br>- 20-s push-ups + 10-s recovery<br>3 min cool-down
	3	3 min warm-up<br>- 8 sets of<br>- 20-s TRX chest press + 10-s recovery<br>- 8 sets of<br>- 20-s TRX squats + 10-s recovery<br>- 8 sets of<br>- 20-s mountain climbers + 10-s recovery<br>- 8 sets of<br>- 20-s skippings + 10-s recovery<br>- 8 sets of

	- 20-s crunches + 10-s recovery<br>- 8 sets of<br>- 20-s stand up, lay down + 10-s recovery<br>3 min cool-down
4	3 min warm-up<br>- 8 sets of<br>- 20-s mountain climbers + 10-s recovery<br>- 8 sets of<br>- 20-s TRX chest press + 10-s recovery<br>- 8 sets of<br>- 20-s skippings + 10-s recovery<br>- 8 sets of<br>- 20-s burpees + 10-s recovery<br>- 8 sets of<br>- 20-s crunches + 10-s recovery<br>- 8 sets of<br>- 20-s TRX squats + 10-s recovery<br>3 min cool-down
